# Efficacy of photodynamic therapy in the treatment of port wine stains: A systematic review and meta-analysis

**DOI:** 10.3389/fmed.2023.1111234

**Published:** 2023-02-21

**Authors:** Ling Wang, Lei Li, Chao Huang

**Affiliations:** ^1^Department of Stomatology, Hospital of Chengdu Office of People’s Government of Tibetan Autonomous Region (Hospital.C.T.), Chengdu, Sichuan, China; ^2^The Clinic of Li Hongjun, Mianyang, Sichuan, China; ^3^Department of Orthopedics, West China Hospital of Sichuan University, Chengdu, Sichuan, China

**Keywords:** photodynamic therapy, port wine stains, clinical assessment, clinimetric study, capillary malformations

## Abstract

**Background:**

Port wine stains (PWS) often cause cosmetic effects and psychological distress. Pulsed dye lasers (PDL) and photodynamic therapy (PDT) are the most commonly used treatments. PDL is still the “gold standard” of therapy to date. However, its shortcomings have become apparent as clinical applications have increased. PDT has been proven as an alternative to PDL. Patients with PWS still lack enough evidence about PDT to make informed treatment decisions.

**Objective:**

The purpose of this systematic review and meta-analysis was to assess the safety and effectiveness of PDT for PWS.

**Methods:**

The online datasets, comprising PubMed, Embase, Web of Science, and the Cochrane Library, were searched for meta-analysis-relevant publications. Two reviewers separately evaluated the risk of bias in each listed study. Grading of Recommendations Assessment, Development, and Evaluation (GRADE) was used to assess the treatment and safety outcomes.

**Results:**

Our search retrieved 740 hits and only 26 studies were finally included. Among the 26 studies included, 3 were randomized clinical trials, and 23 were prospective or retrospective cohort investigations. Based on a gathered assessment, the percentage of individuals achieving a 60% improvement was estimated to be 51.5% [95% confidence interval (CI): 38.7–64.1; *I*^2^ = 83.8%] and a ≥75% improvement was 20.5% (95% CI: 14.5–26.5; *I*^2^ = 78.2%) after 1–8.2 treatment sessions (GRADE score: very low). Due to the statistical diversity of the meta-analysis, a subgroup assessment was performed to determine the sources of diversity. The collected findings indicated that the impact of PDT on enhancing the medical effectiveness of PWS was significant in different treatment sessions, different types of ages, different locations of PWS, and different types of PWS. Pain and edema occurred in most patients. Hyperpigmentation was present in 7.9–34.1% of the patients in 17 studies. Photosensitive dermatitis, hypopigmentation, blister, and scar were infrequently reported, with 0–5.8% incidences.

**Conclusion:**

Photodynamic therapy is recommended as a safe and effective treatment for PWS based on the current evidence. However, our findings are based on poor-quality evidence. Therefore, comparative investigations of a large scale and high quality are necessary to support this conclusion.

## 1. Introduction

Port wine stains (PWS) are a kind of congenital capillary malformation caused by differentiation-impaired endothelial cells with progressive dilation of venule-like capillaries (1, 2). This type of lesion affects 0.3–0.5% of the population, manifests as pinkish spots that expand and darken to varied degrees of hypertrophy or nodule development proportionally to time, and can cause profound cosmetic effects and psychological distress (3–5).

Currently, pulsed dye lasers (PDL) and vascular-targeted photodynamic therapy (PDT) are the most prevalent used therapies. PDL has been used to treat PWS for many years by eradicating capillary abnormality using selective photo-thermolysis, remaining the “gold standard” therapy to date. The shortcomings of PDL therapy are becoming apparent as clinical applications have increased. Up to 20% of patients with PWS are barely lightened with PDL, and recurrence is common after treatment (6–8).

As a promising alternative treatment for PWS, Jiang et al. (9) first used PDT in 1991 in China. With accumulating clinical evidence, PDT has become an alternative to PDL (10–12). This treatment is based on a photochemical interaction that uses photosensitizers combined with light to destroy target tissues leading to apoptosis and endothelial destruction, ultimately leading to thrombosis and vascular occlusion (10).

To date, PDT remains unascertained to assist individuals with PWS in making therapeutic choices (13). This systematic review aimed to improve the therapeutic decision-making process by systematically comparing the data on PDT to treat PWS.

## 2. Methods

### 2.1. Search approach and eligibility standards

We searched PubMed, Embase, Web of Science, and the Cochrane Library from the index date of every database to June 2022 utilizing the following phrase: “Photochemotherapy,” “photochemotherapy,” “photodynamic,” “photochemo,” “phototherapy,” “PDT,” “Photosensitizing Agent* or Photosensitising Agent*,” and “photodynamic therapy* or light therapy*” which were combined with the following words: “port wine stains,” or “naevus flammeus.” The search was restricted to published human studies written in English.

### 2.2. Study selection

Only studies having participants diagnosed with PWS were included, and photodynamic treatment as a PWS therapy was searched. EndNote X9 was used to enter and deduplicate the retrieved articles. Two researchers (LL and CH) selected the included studies independently. To resolve any disagreement, a third reviewer (LW) was consulted.

### 2.3. Inclusion criteria

English-language publications that met the following criteria were included: (i) relevance: original studies of any design that investigated the treatment of PWS with PDT, and (ii) participants: patients of both genders of any age with a clinical diagnosis of PWS.

### 2.4. Exclusion criteria

Exclusion criteria were: (i) other vascular malformation, vessel-related syndromes, or other skin diseases that may interfere with the research evaluation, (ii) acquired/traumatic PWS or another therapy-resistant PWS, and (iii) case reports involving less than five individuals, conference abstracts, *in vitro* studies or animal studies, and non-exclusive PDT treatment.

### 2.5. Outcome measures

#### 2.5.1. Primary outcomes

Efficacy is the Main result, defined as any quantifiable enhancement to the PWS, including clearance, fade, and improvement reported as percentage ranges or changes in the erythema index.

#### 2.5.2. Secondary outcomes

Secondary outcomes included patient satisfaction (Patient Satisfaction Score 0–10: 0, very dissatisfied to 10, very satisfied or graded as “excellent,” “good,” “moderate,” or “ineffective”) and adverse effects.

### 2.6. Information extraction and evaluation of bias risk

Data were extracted, and the Effective Public Health Practice Project (EPHPP) tool was used to independently assess the risk of bias in each included study by two review authors (LL and CH) (14). Disagreements were settled through conversations with the review committee. Whenever required, the corresponding author was contacted for clarification or more information.

### 2.7. Data analysis

Data were analyzed in Stata 17.0 (Stata Corps, STATA 17 Software). The extracted data were presented as means with standard deviations, medians with interquartile ranges, or percentages with 95% confidence intervals (CI). A random-effects model was used to analyze proportions or means when studies showed similar characteristics of patients and treatments.

To facilitate meta-analyses, the various outcome measures were employed to enhance the PWS transformed into dichotomous scales: “excellent efficacy” ≥75% enhancement and “great efficacy” ≥60% enhancement. Accordingly, studies must report their results in terms of quartiles of percentage lightning (such as 0–25%, 25–50%, 50–75%, and 75–100% improvement or 0–20%, 20–60%, 60–90%, and 90–100% improvement). Studies with % ranges that might be translated to classified scales and other percentage ranges were included in the meta-analysis.

## 3. Results

### 3.1. Study characteristics

**FIGURE 1:**
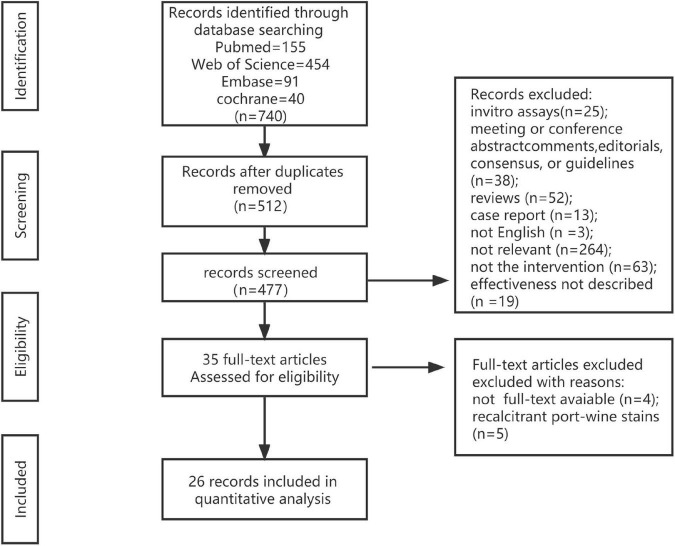
Flow chart for study inclusion.

A total of 740 results matched the current search criteria. According to the title and abstract of the studies, we screened 512 studies after eliminating duplicates. Then, 26 studies were chosen after reading the full texts of 35 articles (11, 12, 15–38) (Figure 1).

Of the 26 studies, 3 were randomized medical trials, and 23 were prospective or retrospective cohort trials (Table 1). In each study, 8–439 patients participated, ranging in age from 1 to 65 years. Most individuals with Fitzpatrick skin types III–IV had PWS ranging from pink to red and even purple. The characteristics of treatment varied between studies. For example, three distinct kinds of photosensitizers were utilized: hematoporphyrin monomethyl ether (HMME, Fudan Zhangjiang Bio Pharmaceutical, Shanghai, China), photocarcinorin (PsD-007, The Second Military Medical University, Shanghai, China or Shanghai Institute of Red-Green photosensitizers, Shanghai, China), or Aminolevulinic acid (ALA, Crawford Pharmaceuticals, Milton Keynes, United Kingdom). Moreover, the total number of therapies (1–8.2), therapy interval (4 weeks to 2–3 months), and follow-up (2 months to 5 years) differed significantly.

**TABLE 1:** Characteristics of included studies (*n* = 26).

References	Country	Study design	Number of subjects	Age (years)	Fitzpatrick skin-type	Location of PWS	Type of PWS	Type of photosensitizers	Photosensitizers dose (mg/kg)
Evans et al. (15)	United Kingdom	PC	8	19–42	-	Leg, trunk	–	ALA	30
Qin et al. (16)	China	RS	238	2–56	–	Face, neck, arm and leg	Pink, flat; light red, flat; dark red, flat; purple, slightly thicker;significantly thicker or nodular	PsD-007	4–5
Yuan et al. (17)	China	RAC	306	3–30	–	Temple, cheek, neck	Color: pink and purple Hypertrophy: no Size: 5–78 cm^2^	HMME	3.5–5
Lu et al. (18)	China	PC	75	5–42	–	Forehead and temple, pars zygomatica, pars buccalis, cervical part, extremities	Pink or bright red, purple	PsD-007	5
Xiao et al. (19)	China	RS	282	11.5–26.3	III–IV	Face and neck, torso, upper limbs, lower limbs	Nodules and papules	HMME	3.5–5
Zhao et al. (20)	China	PC	20	16–50	–	–	Pink or purple	HMME	5
			20						
Gao et al. (21)	China	PC	15	11–36	III–IV	Neck, upper arm, upper leg	Red or purple	HMME	4.5
Ren et al. (22)	China	PC	8	10–46	-	Face and neck	Purple	PsD-007	4–5
Zhang et al. (23)	China	RS	132	3–10	–	Forehead, cheek, Jaw, nose, canthus, upper lip	Red, purple	HMME	3.5
Zhao et al. (24)	China	RCT	329	14–65	III–IV	Non-centrofacial, centrofacial, neck, other	Pink, purple, hypertrophic	HMME	5
Tang et al. (25)	China	RCT	38	15–47	–	–	–	HMME	5
Zhang et al. (26)	China	RS	16	1.5–28	–	Face, neck, forearms and fingers	Pink, purple, thickening	HMME	5
Gan et al. (27)	China	PC	82	1–14	–	Head or neck, trunk, arm, or leg	Pink, purple	HMME	5
Wu et al. (28)	China	RCT	40	16–50	–	–	Pink, purple, hypertrophic	HMME	2.5
			40						5
Ma et al. (29)	China	RS	21	16–32	III–IV	Face or neck	Pink or bright red, purple, hypertrophy	PsD-007	5
			17	14–44				HMME	
Wen et al. (30)	China	PC	22	3–39	-	Face and neck	Pink, purple, thickening	HMME	5
Khalaf et al. (31)	China	PC	45	6–37	III–IV	Cheek, frontotemporal area, lower jaw, middle of the face, and other parts of the body	Pink, purple red, thickened	HMME	5
Li et al. (32)	China	PC	62	2–55	–	Scalp and neck, face, limbs	Pink, purple, nodular thickening	HMME	5
Huang et al. (33)	China	RS	212	1–51	III–IV	Face	Pink, purple, hypertrophic	HMME	5–7.5
Tan et al. (11)	China	RS	439	1–14	–	Face, neck, trunk and extremities	Pink, red, purple	HMME	5
Han et al. (34)	China	RS	11	1–23	III–IV	Face	–	PsD-007	4–7
			2					HMME	5
Li et al. (35)	China	RS	33	4–54	–	Face	Pink, red, purple, dark purple	HMME	5
Lin et al. (36)	China	PC	211	1–58	III–IV	Face and neck	–	HMME	5
Peng et al. (37)	China	RS	39	20.54 ± 13.03	–	Face	Red, purple	HMME	5
Zhang et al. (12) (A)	China	RS	72	18–55	III–IV	Face, neck, and extremities	Purple flat, purple hypertrophic, nodular thickening	HMME	5
Zhang et al. (38) (C)	China	RS	216	1–14	III–IV	Face, neck, arm, or leg	Pink, purple	HMME	5
**Light source**	**Wave length (nm)**	**Energy density (J/cm^2^)**	**Irradiation time (min)**	**Treatment sessions**	**Treatment interval**	**Follow up**	**Primary outcome (quantitative improvement)**	**Secondary outcomes (adverse events)**	
PDL	585	6.5	–	3	4 weeks	2 months	Reduction of lesional redness	Pain, crusting, and bruising	
Copper vapor laser	510.6 and 578.2	160–260	40–60	1–4	2–3 months	6 months to 4 years	Cosmetic improvement	Hyperpigmentation, blistering, peeling, and itching	
Copper vapor laser	510.6 and 578.2	140–240	20–40	1–5	2 months	5 months to 2.5 years	Cosmetic improvement grades	Hyperpigmentation, hypopigmentation, and scarring	
Copper vapor laser	510.6 and 578.2	160–360	30–60	1–4	3 months	3 months	Cosmetic improvement	Hyperpigmentation, Hypopigmentation	
Copper vapor laser	510.6 and 578.2	140–240	20–40	2.6–8.2	2 months	1–5 years	Improvement in% ranges	Pigmentary abnormalities, scarring, eczema dermatitis, and photosensitivity reaction	
Nd: YAG laser	532	96–120	20	1	–	8 weeks	PWS fading in% ranges	Red swelling, blister, pain, burning sensation and crust (at the treated site); edema (around the treated site)	
		144–180	30						
Copper vapor laser	510.6 and 578.2	120	20	1	–	2–8 months	Blanching rate	Pigmentation, atrophy, scar formation, and infection	
Nd:YAG laser	532	96–180	20–30	–	–	3–6 months	Lightening in%	–	
Copper vapor laser	510.6 and 578.2	80–100	20–25	1	–	2 months	Blanching in% ranges	Hyperpigmentation, hypopigmentation, and scarring	
Nd: YAG laser	532	96–120	20	2	8 weeks	16 weeks	PWS fading in% ranges	Burning sensation, pain, pruritus, numbness, edema, purpura blistering, and crusting	
IDAS	532	48–60	10	2	8 weeks	16 weeks	–	Pain, Scab, blisters, pruritus, swelling, and burning sensation	
LED light	532	96–142.5	20–25	1	–	2 months	PWS fading in% ranges	Burning sensation, pain, edema, hyperpigmentation	
LED light	532	96–115	20	2	8 weeks	–	Improvement in%	Pain, burning sensation, edema, purpura, crust, hyperpigmentation, and hypopigmentation	
Nd: YAG laser	532	96–120	20	2	8 weeks	16 weeks	PWS fading in% ranges	Photosensitive reactions, hyperpigmentation, hypopigmentation, scab and infection	
KTP laser	532	96–108	20–30	1	–	2 months	Blanching in% ranges	Edema, pruritus, scabs, blisters, scars, hyperpigmentation, or hypopigmentation	
LED light	532	96–180	20–30	1	–	8 weeks	Blanching in% ranges	–	
LED light	532	80–110	15	3	–	–	Lesion subsided in% ranges	Pain, redness, swelling, light brown crusts, hyperpigmentation, and scarring	
LED light	532	96–180	20–30	2	2 months	4 months	Clearance degree in% ranges	Edema, crust, hyperpigmentation, blister, eczema, scar	
LED light	532	86.4–150	18–25	1–6	2 months	–	Improvement in% ranges	Burning, pain, itchiness, edema, crusting, blistering, hyperpigmentation, hypopigmentation, infection, and scarring	
LED light	532	96–115	20–25	–	8 weeks	8 weeks	Improvement in% ranges	Pain, swelling, purpura, crusting, eczema, pigmentation, or scarring	
Nd:YAG laser	532	120–162	25–30	4.38 ± 4.907	–	7.1 ± 4.34 years	Improvement in% ranges	–	
		96–162	20–30						
LED light	532	105–120	20–25	–	2–6 months	–	Improvement in% ranges	Cutaneous adverse events, light-exposure related adverse events, systemic adverse events	
LED light	532	96–120	20	–	2–3 months	–	Improvement in% ranges	Edema, pruritus, pain, small blisters, scab, pigmentation, hypopigmentation, infection, scar, photosensitive reaction, systemic symptoms	
LED light	532	96–120	20–30	2	2–6 months	3 months	PWS fading in% ranges	Pigmentation, hypopigmentation, depigmentation, and scar formation	
LED light	532	102–127.5	20–25	2	2 months	≥1 years	PWS fading in% ranges	Burning sensation, pain, pruritus, edema, purpura, crust, urticaria	
LED light	532	84–120	20–25	2	2 months	≥1 years	PWS fading in% ranges	Burning sensation, pain, pruritus, numbness, edema, purpura, blistering, crusting, hyperpigmentation, and hypopigmentation	

cm^2^, square centimeter; HMME, hematoporphyrin monomethyl ether; IDAS, ist das leistungsstarke, transportable LBO-laser system; J/cm^2^, joule per square centimeter; KTP laser, potassium titanyl phosphate laser; LED light, light emitting diode; Mg/kg, milligrams per kilogram; min, minute; Nm, nanometer; Nd:YAG laser, neodymium-doped yttrium aluminum garnet laser; PC, prospective cohort; PDL, pulsed dye laser; PsD-007, photocarcinorin; PWS, port wine stain; RAC, retrospective analytic cohort; RCT, randomized controlled trial; RS, retrospective study; –, not reported. Zhang et al. (12) (A), subjects: adult patients; Zhang et al. (38) (C), subjects: children patients.

### 3.2. Evaluation of bias risk

Using the EPHPP tool, we determined that 23 out of 26 non-randomized experiments were of poor quality. Three randomized controlled trials (RCT) provided moderate evidence due to the absence of adjustment for confounding parameters (age, gender, PWS type, and PWS location) in the study design and the use of non-validated and unreliable results measuring tools.

### 3.3. Treatment outcomes

**FIGURE 2:**
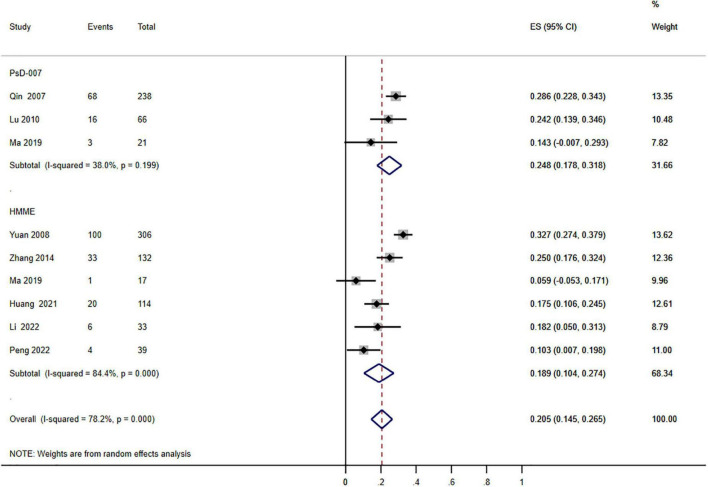
Forest plot of the effects of photodynamic therapy (PDT) according to different types of photosensitizers (≥75% improvement). Weights are from random effects analysis.

**FIGURE 3:**
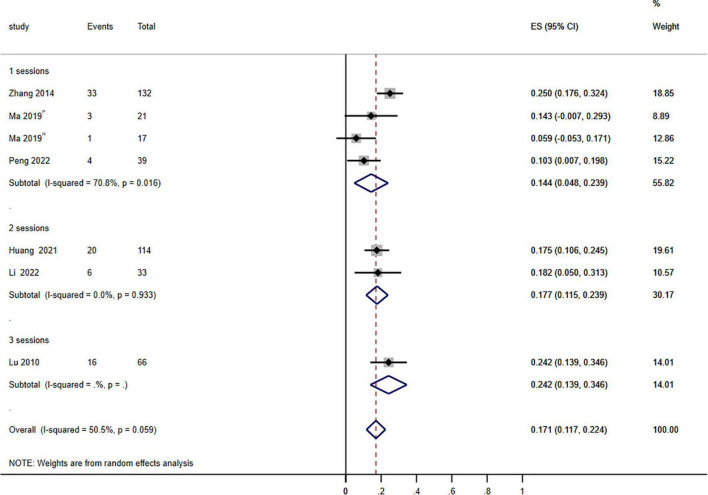
Forest plot of the effects of photodynamic therapy (PDT) on treatment sessions (≥75% improvement). P: PsD-007-PDT; H: HMME-PDT. Weights are from random effects analysis.

**FIGURE 4:**
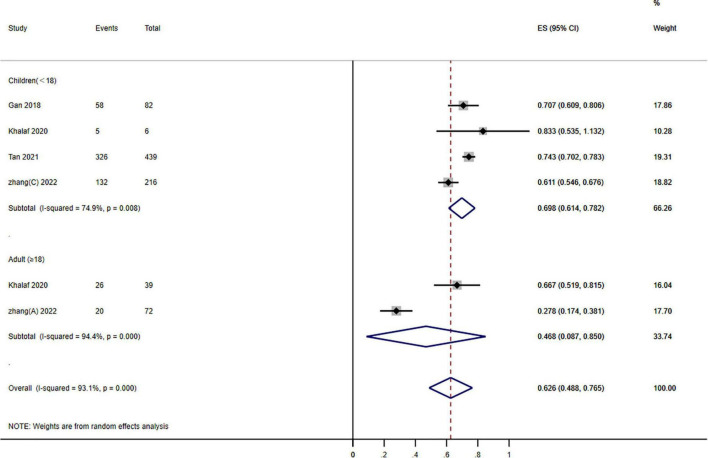
Forest plot of the effects of photodynamic therapy (PDT) according to different age groups of port wine stains (PWS) (≥60% improvement). Weights are from random effects analysis.

**FIGURE 5:**
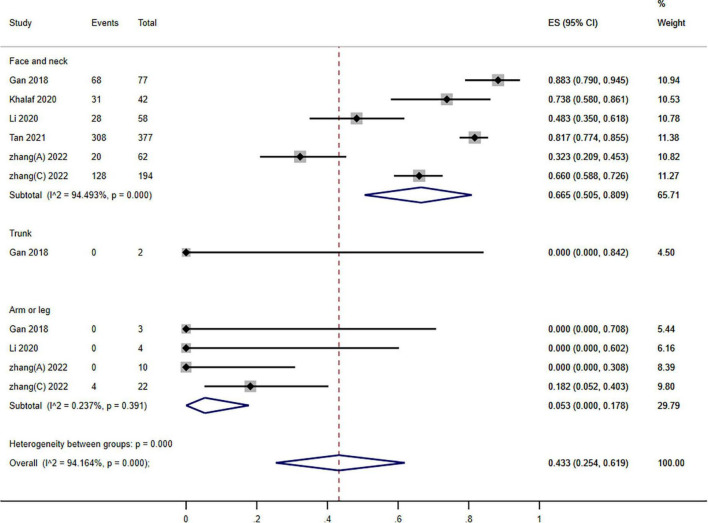
Forest plot of photodynamic therapy (PDT) effects according to different locations of port wine stains (PWS) (≥60% improvement).

**FIGURE 6:**
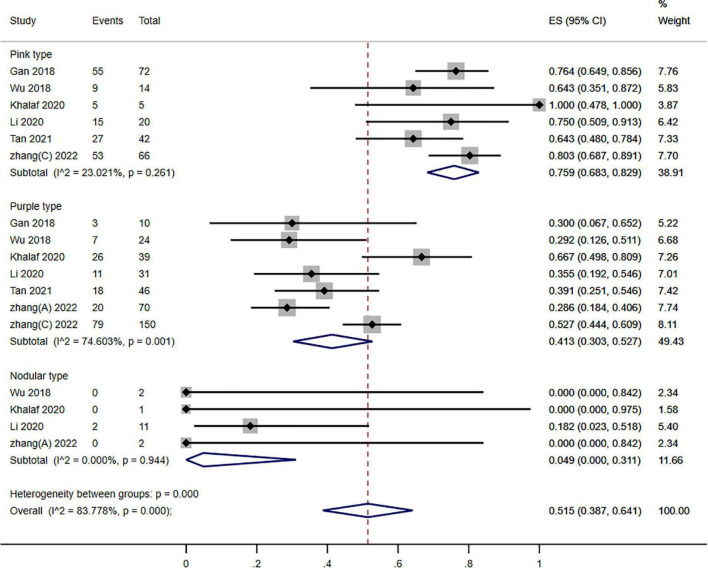
Forest plot of photodynamic therapy (PDT) effects according to different types of port wine stains (PWS) (≥60% improvement).

The therapeutic regimen summed up the findings of the individual trials (Figures 2–5). Due to the heterogeneity of outcomes, the small number of trials documenting our results prevented the pooling of the majority of outcomes. Moreover, 26 investigations on PDT with 3,034 individuals were conducted. According to a pooled estimate, 51.5% of individuals showed an improvement of ≥60%, [95% confidence interval (CI) 38.7–64.1; *I*^2^ = 83.8%] (Figure 6) and a ≥75% improvement was 20.5% (95% CI 14.5–26.5; *I*^2^ = 78.2%) (Figure 2) after 1–8.2 therapeutic sessions with various photosensitizers and lighting sources. Accordingly, the randomized impact model was chosen due to the high statistical heterogeneity in our findings. Consequently, a subgroup assessment was conducted to determine the origins of diversity, stratified into different types of photosensitizers, different treatment sessions, different types of ages, different locations of PWS, and types of PWS.

Subgroup analysis stratified by different photosensitizer kinds (PsD-007 and HMME): the aggregated results based on the various photosensitizers showed that between 14.5 and 26.5% of the patients achieved excellent efficacy (≥75% improvement), and significant heterogeneity was detected (*P* = 0.000; *I*^2^ = 78.2%). Nevertheless, a subgroup distinction was absent between the two kinds of photosensitizers (*P* = 0.289; *I*^2^ = 1.123) (Figure 2).

To further understand efficacy within various treatment sessions of PDT for PWS, we evaluate the clinical efficacy of PDT after 1–3 sessions. The pooled data demonstrated that the impact of PDT on the therapeutic effectiveness of PWS was statistical significance in different treatment sessions (*P* = 0.044; *I*^2^ = 6.228) (Figure 3).

Subgroup analysis stratified groups according to age: children (<18 years) and adults (≥18 years). The outcomes showed that PDT enhanced therapeutic effectiveness: 48.8–76.5% of patients have a ≥60% improvement. A significant subgroup variation was found between the various age groups (*P* = 0.000; *I*^2^ = 23.060) (Figure 4).

Subgroups analysis stratified groups according to the various PWS sites: the pooled assessment of the number of individuals who improved by ≥60% was 43.3% (95% CI: 25.4–61.9; *I*^2^ = 94.164%). Nonetheless, we observed a significant subgroup difference among different locations (*P* = 0.000; *I*^2^ = 30.227) (Figure 5).

To conduct stratified subgroup analysis according to the various kinds of PWS, individuals were separated into the 3 kinds of PWS (pink, purple, and nodular). The gathered outcomes of various kinds of PWS demonstrated that 75.9, 41.3, and 4.90% of the patients reached a ≥60% improvement in pink, purple, and nodular types of PWS, respectively, and showed a significant variation (*P* = 0.000; *I*^2^ = 83.778%). Nevertheless, significant subgroup differences existed across the three kinds of PWS (*P* = 0.000; *I*^2^ = 40.900) (Figure 6).

### 3.4. Overall quality of the evidence

The overall quality of the evidence (GRADE) was rated as very low (Supplementary Table 1). High bias risks in result measures and publication bias, non-validated outcome measuring equipment, comparatively smaller sample sizes (imprecision), inadequate data, and the variability of therapeutic response were mostly the causes.

### 3.5. Satisfaction

Among the 26 studies, five of them generally reported patient satisfaction. There was a 94.67% patient satisfaction rate in Lu et al.’s (18) study. According to Xiao et al. (19), PDT was generally well received by patients (patient satisfaction score 7.8). Moreover, three studies reported that the rate of patients graded as “excellent” was 17.5–35%, and the rate of patients graded as “good” to “excellent” was 60–95% (20, 24, 28).

### 3.6. Adverse events

Overall, few adverse events were documented and none were serious (Table 2). Nevertheless, most individuals had pain and edema, and Zhao et al. (20) and Tang et al. (25) reported that most of these AEs were moderate in severity (Supplementary Figures 1, 2). Hyperpigmentation was present in 7.9–34.1% of patients in 17 studies (Supplementary Figure 3). Other AEs included Photosensitive dermatitis (0–2.6%), hypopigmentation (0.2–2.2%), scar (0.4–2.3%), and blister (0.6–5.8%).

**TABLE 2:** Adverse events.

References	Hyperpigmentation		Hypopigmentation		Scar		Pain		Photosensitive dermatitis		Edema		Blister	
	**Events**	**Total**	**Events**	**Total**	**Events**	**Total**	**Events**	**Total**	**Events**	**Total**	**Events**	**Total**	**Events**	**Total**
Qin et al. (16)	172	238	–	–	–	–	–	–	1	238	–	–	1	238
Yuan et al. (17)	22	306	3	306	13	306	–	–	2	306	–	–	–	–
Lu et al. (18)	7	75	1	75	–	–	–	–	–	–	–	–	–	–
Xiao et al. (19)	22	507	6	507	11	507	–	–	3	507	–	–	7	507
Zhao et al. (20)	13	20	4	20	2	20	15	20	4	20	18	20	2	20
Gao et al. (21)	0	15	0	15	0	15	–	–	0	15	15	15	–	–
Zhang et al. (23)	5	132	2	132	4	132	–	–	–	–	–	–	–	–
Tang et al. (25)	–	–	–	–	–	–	34	35	–	–	27	35	5	35
Zhang et al. (26)	4	16	–	–	–	–	7	16	–	–	15	16	–	–
Gan et al. (27)	20	82	2	82	–	–	80	82	–	–	77	82	–	–
Wu et al. (28)	23	50	1	50	–	–	–	–	4	50	–	–	–	–
Ma et al. (29)	0	38	0	38	0	38	38	38	–	–	38	38	0	38
Khalaf et al. (31)	4	45	–	–	0	45	45	45	–	–	–	–	–	–
Li et al. (32)	4	62	–	–	1	62	–	–	–	–	52	62	1	62
Huang et al. (33)	26	212	6	212	5	212	–	–	–	–	193	212	13	212
Tan et al. (11)	294	439	–	–	9	439	417	439	–	–	–	–	–	–
Lin et al. (36)	61	211	0	211	0	211	128	211	0	211	211	211	10	211
Peng et al. (37)	6	39	1	39	1	39	–	–	–	–	–	–	–	–
Zhang et al. (12) (A)	–	–	–	–	–	–	72	72	–	–	72	72	–	–
Zhang et al. (38) (C)	–	–	–	–	1	216	–	–	–	–	216	216	–	–

## 4. Discussion

Our systematic review discusses 26 trials wherein PDT was administered to 3,034 patients with PWS. Quantitative recovery of PWS lesions and documented negative impacts differed significantly among the trials and therapeutic regimens. Our systematic review aimed to assess the efficacy and safety of PDT for individuals with PWS. This evidence is essential for patients with PWS to make treatment decisions. HMME-PDT was frequently used, and 51.5% of the patients showed a ≥60% improvement. Patients were well responsive to and satisfied with PDT.

Generally, adverse effects were transient and no special intervention was required. Pain and edema occurred commonly following PDT in kids and adults; However, only two studies assessed pain severity. Significantly, a mild correlation coefficient was detected between pain severity at 5 min and treatment efficiency in Tang et al.’s (25) study. It has been noted that photosensitivity is one of the major complications of PDT (17, 36). Therefore, sunlight light exposure of skin and eyes should be avoided within 2 weeks. Hyperpigmentation and hypopigmentation would fade within 2–6 months (16, 27). The formation of scars was probably caused by dermal overdose, as well as the thermal damage to the dermis.

Port wine stains usually appear at birth and get progressively darker and thicker with age. PDL and PDT are the mainstays in treating PWS. Evans et al. (15) suggested that HMME-PDT is at least as effective as 585 nm PDL in terms of blanching and adverse effects. Four studies analysis demonstrates that the therapeutic effect of PDT was better than that of PDL therapy under the different evaluation criteria (17, 21, 23, 37). Significantly, PDT is more effective than PDL for purple flat lesions (17, 23, 37). However, PDL is more appropriate for younger patients and superficial lesions and unsuitable for patients with nodular lesions or Fitzpatrick skin types IV or V. There was a wide range of side effects reported in different studies regarding PDT and PDL. Compared to PDT, Yuan et al. (17) and Zhang et al. (23) found PDL have more side effects and the children PDT group showed the lowest side effects rate (1% hyperpigmentation and 3% scars), possibly attributed to a lower dose of PDT. It is likely that this was due to a lack of dynamic cooling technique during PDL which could offer better efficacy and fewer side effects. In Peng et al.’s (37) study, there existed no significant difference in the incidence of side effects between different types of PWS in PDT and PDL group. Overall, compared with PDL, PDT might be an alternative approach for treating these patients and reaching a great therapeutic effect with few side effects.

With the photosensitizer, PDT could destroy vessels of any size in PWS. HMME, the most commonly used photosensitizer, was launched into the Chinese market and applied in the clinic in 2017 with a stronger photodynamic effect, fewer side effects, and a higher selective effect on abnormal vascular endothelial cells (26). HMME quickly reaches peak concentration in the blood after intravenous injection and is quickly absorbed by abnormal endothelial cells. The next step is irradiating a specific wavelength of light, which generates singlet oxygen and other reactive oxygen species. It selectively destroys the deformed HMME-containing capillary network without causing damage to normal tissues. Through years of clinical observation, HMME-PDT has been affirmed as a viable option for PDL in China for the management of PWS due to its proven effectiveness and safety (35).

Photodynamic therapy efficacy might vary based on sex, age, PWS location, PWS type, PDT treatment parameters, and treatment sessions, presumably due to differences in the lesion-involved skin and vessel properties (19, 39). Similar results were found in our subgroup analysis, validating our research.

Zhao et al. (24) suggest that multiple treatment sessions application may be preferable for PWS due to high satisfaction rates from treating physicians and patients in the PDT group. Furthermore, individuals who received multiple PDT sessions displayed a cumulative response (11). Although photosensitizer has a powerful, damaging impact on abnormal blood vessels, only a limited amount of efficacy can be attained in a single therapeutic session. Patients with severe PWS lesions commonly require multiple treatment sessions to eliminate the abnormal capillary network and prevent a recurrence.

Age was associated with an elevation in PWS lesion colors, thickness, formation of nodules, and change from pink to purple. Accordingly, age contributes to PWS progression and PDT response. The younger the patient, the greater the treatment response because PWS lesions are at their thinnest. The earliest possible treatment of PWS could prevent physical and psychological complications (23). Additionally, an extended follow-up study is required to determine the optimal age to begin therapy. However, there are some different viewpoints. Lin et al. (36) found a strong association between age and the kind of PWS lesion. Increasing age causes the port-wine stain lesions to thicken and darken, diminishing the efficacy of treatment. Accordingly, PWS lesion type was considered an irrelevant factor associated with efficacy.

Port wine stains on the face and neck respond more effectively than those on the trunk or limbs (11, 27). It is hypothesized that lesions located with thin skin regress more favorably after treatment since light penetrates more easily into thin skin (27). Meanwhile, observations showed no difference in patients with face, scalp, and neck (23, 32).

Specifically, PDT showed excellent therapeutic effects on patients for pink type PWS than purple type and nodular type. These differences might be explained by the vascular network differences between the three kinds of PWS. Compared to pink-type lesions, purple-type and nodular-type lesions have deeper, wider, and thicker vessels. Moreover, different PWS types may have distinct vascular structures, vascular densities, and skin tissue densities (11). These parameters can influence light penetration and photochemical reaction intensity. Subsequently, difficulties still exist in treating thicker or nodular lesions, and multiple treatments are often required (17).

However, the involved trials in this research were of low to moderate methodological quality. It was primarily due to the infrequent correction of confounding variables and the absence of validated outcome measurement instruments in some studies. Furthermore, heterogeneity was high among and within studies regarding patient characteristics, PWS lesions, and PDT parameters. Accordingly, some lower-quality trials can be pooled, leading to inadequate subgroup analysis due to heterogeneity and small subgroup sizes. Despite this, this review provided the most up-to-date evidence available. Herein, a combination of observational and experimental studies was included, enabling us to gather evidence of treatment safety and effectiveness.

When possible, researchers dichotomized and classified therapeutic results. Multiple trials that used various outcome measures exist. This prevented the pooling of outcomes and valid comparison of available therapeutic options. Several qualitative impacts were turned into quantitative results to compare research results. It is inevitably arbitrary to convert these outcomes, and interpretation should be done cautiously.

Photodynamic therapy for PWS is the focus of this review, whereas previous reviews primarily covered PDL. Moreover, the present trial has shortcomings in the available information regarding PWS treatment effectiveness trials. Due to the lack of uniformity in the outcome of medical trials on PWS, it is challenging to compare the outcomes of clinical trials on PDT. According to this paper, we have acquired more knowledge about which outcomes of patients with PDT are most important. Finally, due to the insufficient documentation of PWS lesion features and treatment outcomes, we concluded that PDT is effective and safe for patients with PWS who need more high-quality clinical research.

## 5. Suggestion for practice

Herein, PDT was recommended to have significant positive effects on PWS. PDT might be utilized to manage individuals with PWS in the clinic. According to our findings, young patients with PWS in the face and neck, pink-type, and multiple treatment sessions responded better to the PDT. Therefore, we will further explore therapy modalities according to the above factors to obtain the most effective medical result and give the best credible evidence for medical care. This research has a few drawbacks. First, the evidence quality is restricted because of the low number of high-quality research and because only three were RCTs. Second, experimental drawbacks, specifically, a large deal of diversity among all included publications. Subgroup analysis was performed to examine the heterogeneity source. High heterogeneity still existed in some groups. Finally, the evaluation is primarily based on the visual assessment. Therefore, objective assessment tools such as chromameters and controlled trials with a larger sample size are necessary to reach more reliable conclusions.

## 6. Conclusion

For patients with PWS, PDT is recommended as a safe and effective treatment based on the current evidence. Our systematic review may assist physicians and individuals in treatment decision-making for patients with PWS. Nevertheless, the current findings depend on evidence of poor to moderate quality. Consequently, large-scale, high-quality comparison research with dependable, validated procedures and standardized outcome data is required.

## Data availability statement

The original contributions presented in this study are included in the article/Supplementary material, further inquiries can be directed to the corresponding author.

## Author contributions

LW designed and supervised the study. LL performed the all databases searches. LL and CH extracted the data. LW and CH conducted qualitative analysis. All authors participated in manuscript writing, contributed to critical comments and revised the manuscript.

## Supplementary material

Supplementary Figure 1 Forest plot of pain.

Supplementary Figure 2 Forest plot of edema.

Supplementary Figure 3 Forest plot of hyperpigmentation.

Supplementary Table 1 Quality assessment of 26 included studies according to the EPHPP tool.
