# Supplementary figures and images for: Efficacy of photodynamic therapy in the treatment of port wine stains: A systematic review and meta-analysis
Source: Front Med (Lausanne). 2023 Feb 21;10:1111234. doi: 10.3389/fmed.2023.1111234 (PMC9988944; doi:10.3389/fmed.2023.1111234)

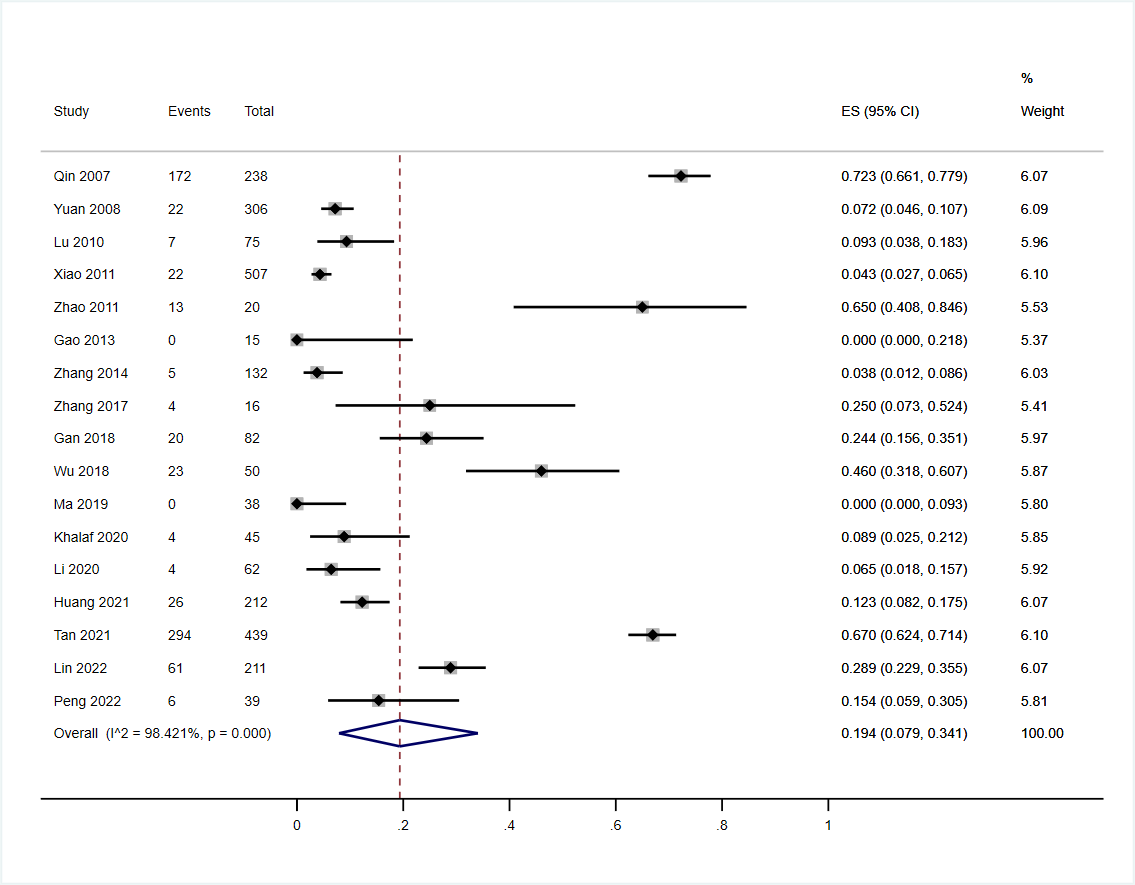

Supplement: Supplementary Figure 1 — Forest plot of pain. [file Image_1.TIF]

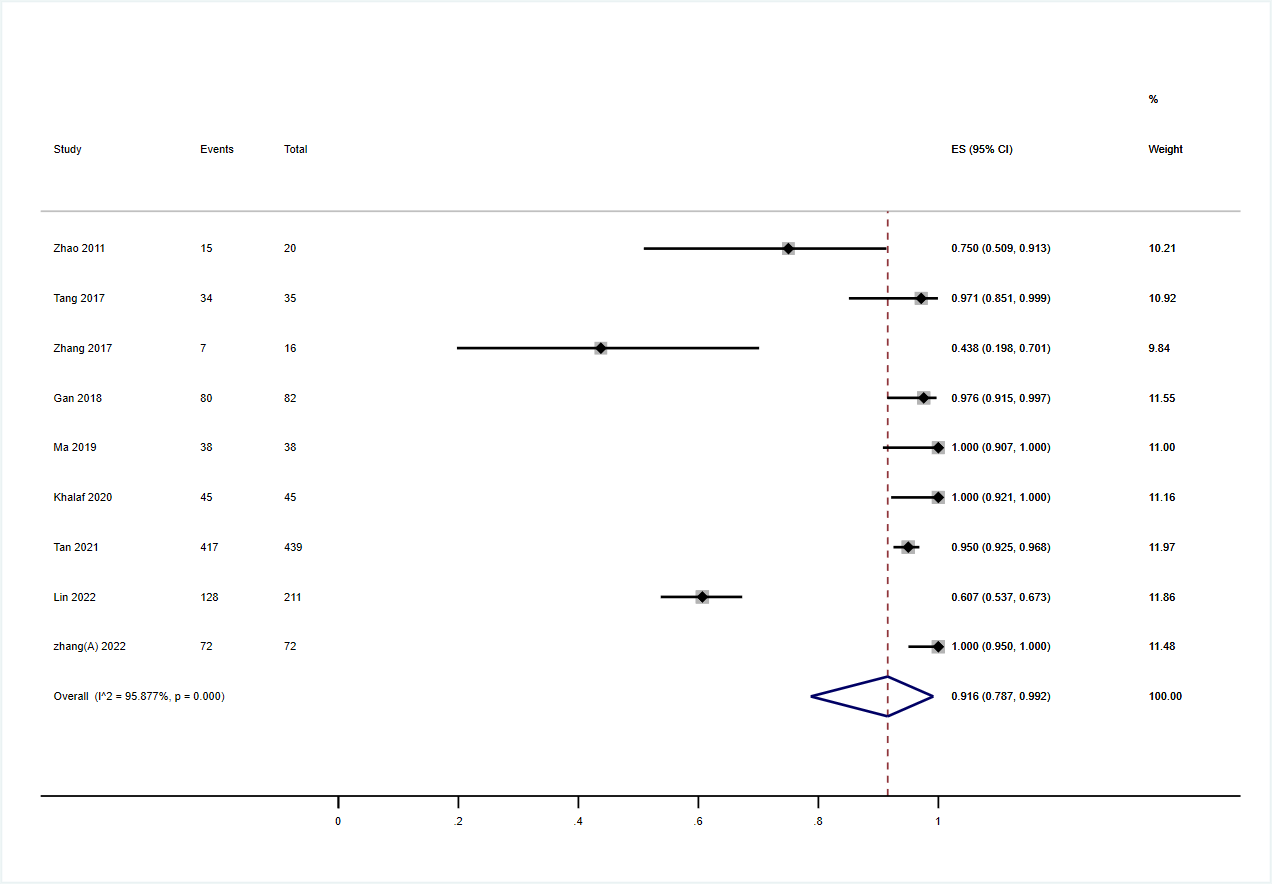

Supplement: Supplementary Figure 2 — Forest plot of edema. [file Image_2.TIF]

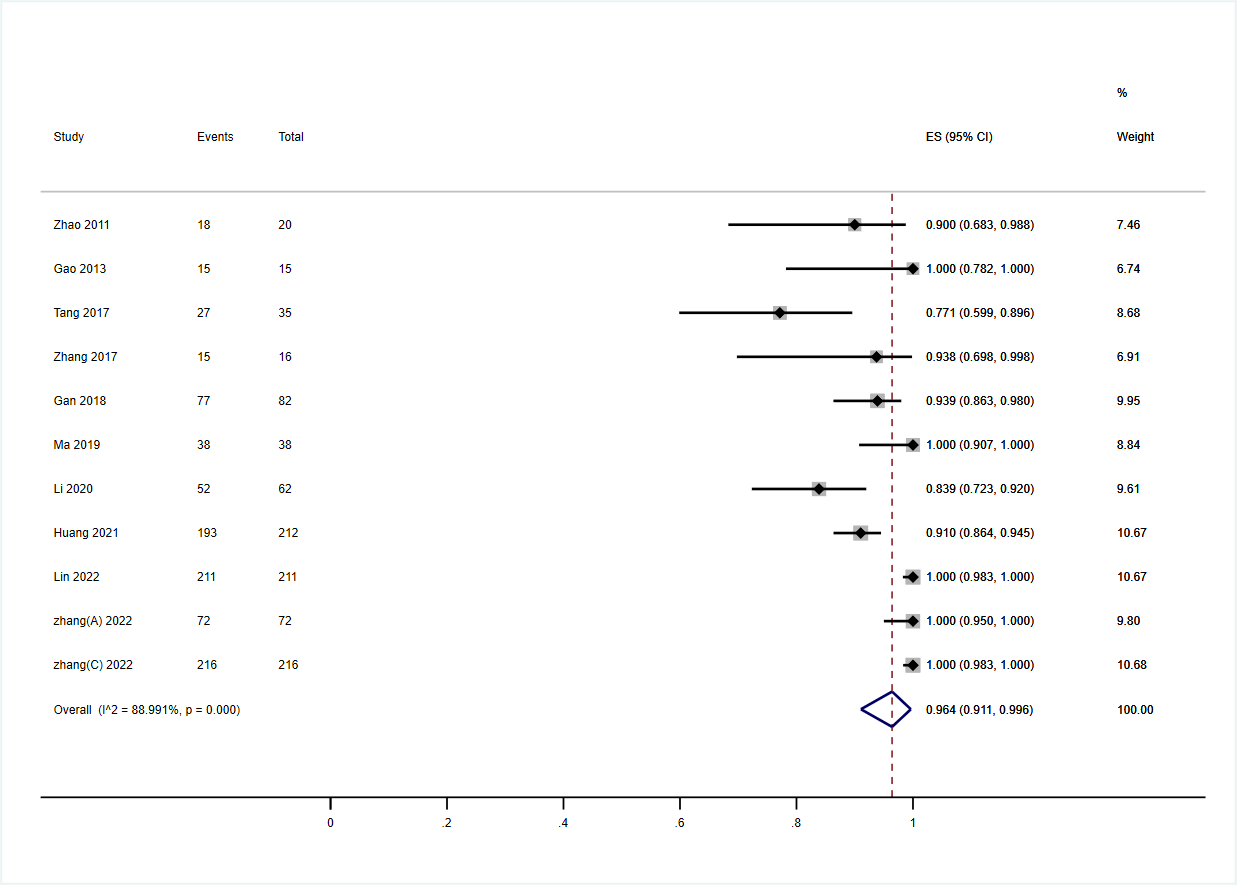

Supplement: Supplementary Figure 3 — Forest plot of hyperpigmentation. [file Image_3.TIF]
